# Glioblastoma Stem Cells as Targets for Emerging Precision Immunotherapies and Molecular Treatments

**DOI:** 10.3390/cells15090783

**Published:** 2026-04-26

**Authors:** Dennis A. Steindler, Katherine Karakoula

**Affiliations:** 1 Steindler Consulting, Boston, MA, USA; 2 Round Table Research, Inc., Research Triangle Park, Chapel Hill, NC 27709, USA; 3 The Eshelman Institute for Innovation, University of North Carolina at Chapel Hill, Chapel Hill, NC 27599, USA; 4 School of Pharmacy and Life Sciences, Research Institute of Healthcare Sciences, University of Wolverhampton, City Campus, Wolverhampton WV1 1LY, UK; a.karakoula@wlv.ac.uk

**Keywords:** neurogenesis, glioma, glioblastoma, cancer stem cell, molecular and immunotherapies, data science

## Abstract

Advances in organoid and other three-dimensional culture systems, single-cell and spatial transcriptomics, multi-omics, and high-resolution imaging are reshaping our understanding of the cellular origins and evolutionary trajectories of glioblastoma. When integrated with modern data science approaches, these technologies enable the construction of increasingly detailed molecular biographies of normal neural stem and progenitor cells as well as malignant stem-like cellular states. Such molecular biographies illuminate how developmental programs, cellular plasticity, and microenvironmental cues are co-opted during gliomagenesis. At the same time, progress in machine learning, immunotherapy, and precision molecular targeting is beginning to translate these biological insights into therapeutic strategies that specifically disrupt glioblastoma stem-like states. Together, these converging approaches provide a conceptual and technological framework for improved tumor modeling, earlier detection, and increasingly personalized therapies for malignant gliomas.

## 1. Introduction: Normal and Pathological Stem Cell Neurogenesis

The concept of persistent cell genesis in the adult brain—“neuropoiesis”—has been debated for more than a century [1]. Early work by Ramón y Cajal argued that the adult mammalian brain lacked regenerative capacity, establishing a long-standing belief that no new neurons were produced after development [2]. This view began to shift in the 1990s with the discovery of neural stem/progenitor cells in the adult rodent and human subependymal zone (SEZ) and hippocampus, demonstrating that limited neurogenesis persists throughout life [3,4,5,6]. These findings launched a modern re-evaluation of adult neurogenesis, supported by studies identifying proliferative progenitors in neurogenic niches of the human brain.

Yet the extent and functional relevance of adult neurogenesis remain controversial. Some studies report robust neurogenesis in the adult hippocampus [4,6], while others describe a sharp decline to near undetectable levels after childhood [7]. Recent single-cell and spatial transcriptomic analyses have revived the debate by identifying rare but persistent neural progenitors even in the aged human hippocampus [8,9,10], suggesting that neurogenesis may be more heterogeneous and context-dependent than previously appreciated.

In contrast, hematopoiesis provides a clear example of lifelong stem cell-driven tissue renewal, highlighting the unique regenerative limitations of the adult brain and heart. While epithelial and mesenchymal tissues maintain substantial regenerative capacity, the central nervous system remains largely refractory to repair following injury or disease [11].

## 2. Stem Cell Pathologies: “Good” and “Bad” Stem Cells

The modern concept of a stem cell originates from pioneering work by Stevens, Martin, Evans, Reynolds, and Weiss, who established the foundations of embryonic and adult stem cell biology [12]. Subsequent studies identified multipotent astrocytic stem/progenitor cells (MASCs) and adult human neural progenitor cells (AHNPs) in the human brain, particularly within the olfactory system and hippocampus [13,14,15]. These populations exhibit stem-like properties but contribute primarily to gliogenesis rather than neurogenesis during adult life. This leads to an important conceptual framework: Adult stem cells can be “good” or “bad,” depending on whether they maintain tissue homeostasis or contribute to pathology.

Normal adult stem cells may support limited gliogenesis during aging, respond to injury with restricted regenerative potential and fail to replace neurons lost to trauma or neurodegenerative disease. Conversely, pathological stem cells may proliferate excessively, acquire mutations, generate aberrant progeny and initiate neoplastic growth.

This duality forms the basis of stem cell pathologies, in which stem/progenitor cells either fail to repair damaged tissue or contribute to hyperplasia and tumorigenesis [16]. The inability of adult neural stem/progenitor cells to restore circuitry lost to stroke, traumatic injury, or neurodegenerative disease (e.g., Parkinson’s disease) exemplifies one form of stem cell pathology [17]. The opposite extreme—excessive proliferation and malignant transformation—underlies the emergence of brain tumors such as glioblastoma.

The same neural stem and progenitor cell populations that support limited adult neurogenesis can, under pathological conditions, become the cells of origin for gliomas. Understanding how normal stem cell biology intersects with malignant transformation requires examining the ontogeny, lineage relationships, and microenvironmental influences that drive gliomagenesis.

## 3. Ontogeny and Cells of Origin of Gliomas

### 3.1. From Normal Stem Cells to Cancer Stem Cells

The cancer stem cell (CSC) concept was first established in hematologic malignancies [18,19], where rare stem-like cells were shown to initiate leukemia following transplantation. This framework was soon extended to solid tumors, including breast cancer—coinciding with the identification of normal mammary stem cells—and subsequently to osteosarcoma, colon cancer, prostate cancer, and gliomas [20,21,22,23,24,25]. In gliomas, multiple groups identified stem-like populations capable of self-renewal, multilineage differentiation, and tumor initiation in xenograft models [22,23,24].

Glioma-initiating cells (GICs) are thought to arise from glial lineage stem or progenitor cells that retain latent proliferative capacity. In growth-permissive microenvironments—such as those shaped by inflammation, injury, or reactive gliosis—these cells may re-enter the cell cycle and accumulate oncogenic mutations. Candidate cells include multipotent astrocytic stem/progenitor cells (MASCs) [1,12,13], adult human neural progenitor cells (AHNPs) [13], vestigial boundary astrocytes in the gray matter and persistently reactive white matter astrocytes [25], and oligodendrocyte precursor cells (OPCs/NG2+ cells). Although these progenitor populations are not able to regenerate neuronal or glial populations—a limitation sometimes described as a “stem cell pathology”—their retained developmental programs and proliferative potential make them susceptible to malignant transformation.

### 3.2. Neurogenic Niches as Tumorigenic Niches

The developmental subventricular zone (SVZ) and the adult subependymal zone (SEZ) [6] are lifelong neurogenic niches enriched in extracellular matrix components, morphogens, and growth factors. Normal neurogenesis in these regions involves periods of rapid proliferation during which neuronal progenitor cells are vulnerable to genetic instability. During differentiation, progenitors may undergo chromosomal instability, including aneuploidy [26], providing opportunities for oncogenic lesions to arise.

This vulnerability is heightened during periods of inflammation, aging, injury, and reactive gliosis, all of which generate microenvironments that promote progenitor activation and cycling. These conditions support the view that gliomagenesis often emerges from endogenous progenitors placed into pathological states of proliferation. OPCs (NG2+ cells), which normally generate oligodendrocytes and astrocytes, have been strongly implicated as cells of origin for IDH-mutant gliomas [14]. Likewise, astrocytic stem/progenitor cells residing in the SVZ/SEZ can give rise to tumors such as subependymal giant cell astrocytoma (SEGA), particularly in the setting of TSC1/TSC2 mutations [27]. The SEZ exemplifies how a lifelong neurogenic niche—rich in proliferative cues—can support the emergence and maintenance of tumorigenic stem-like cells.

The precise origins of IDH-mutant versus IDH wild-type gliomas remain an area of active investigation and debate [28]. It is increasingly clear that gliomas may arise from multiple progenitor pools depending on developmental timing, regional context, and mutational events. Identifying and therapeutically targeting the true cells of origin remain challenging and will require longitudinal human tissue studies, including xenotransplantation of patient-derived CSC populations into rodent “avatar” models [29]. Such approaches allow researchers to observe tumor initiation, evolution, and therapeutic response in vivo, providing critical insights into the earliest events of gliomagenesis.

### 3.3. Lineage Tracing, Clonal Evolution, and Tumor Heterogeneity

Lineage tracing studies in genetically engineered mouse models, including NF1- and PDGF-driven glioblastomas, demonstrate that glial progenitors are capable of initiating high-grade tumors [30]. Genetic barcoding approaches further reveal early clonal extinction, intercellular competition, and dynamic clonal evolution during glioma formation [31]. These processes underlie the profound intratumoral and intertumoral heterogeneity characteristic of glioblastoma.

Recent single-cell RNA sequencing, spatial transcriptomics, and multimodal longitudinal profiling have refined our understanding of glioma ontogeny [32,33,34,35]. Distinct cellular states—including stem-like, mesenchymal, oligodendrocyte-like, and astrocyte-like populations—coexist within individual tumors and shift in response to therapy. Spatial niches such as perivascular and hypoxic regions impose selective pressures that sustain particular GIC states, influence clonal competition, and contribute to therapeutic resistance.

**Figure 1:**
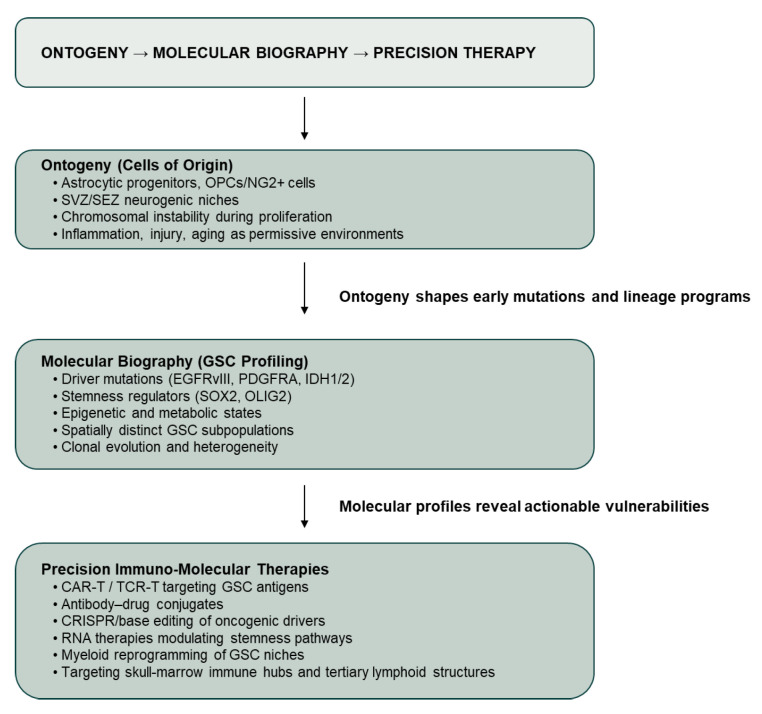
Ontogeny to precision therapy in glioblastoma. Glioblastoma is conceptualized as a progressive transformation from neural ontogeny, through acquisition of a tumor-specific molecular biography, toward therapeutically tractable stem-like states. Cells of origin within astrocytic and oligodendrocyte lineage progenitor pools, shaped by permissive microenvironments, influence early mutational trajectories and lineage programs. Molecular profiling of glioblastoma stem cell (GSC) populations reveals genetic, epigenetic, metabolic and spatial vulnerabilities that inform precision immuno-molecular therapeutic strategies.

Taken together, these findings support a model in which gliomas arise from a restricted set of glial lineage stem and progenitor populations residing within specialized neurogenic or inflammatory niches. Yet identifying the initial cell of origin is only the first step. Translating ontogeny into therapy requires reconstructing the “molecular biography” of glioma-initiating cells: the sequential genetic lesions they acquire, the developmental programs they retain, the microenvironmental cues that maintain them, and the immune interactions that shape their evolution. We propose a conceptual framework in which glioblastoma evolves from neural ontogeny through the acquisition of a tumor-specific molecular biography toward precision immuno-molecular intervention (Figure 1).

## 4. Molecular Biographies of Glioma Cells as a Foundation for Immunotherapy and Precision Medicine

The expanding molecular and cellular “biographies” of glioma stem and progenitor cells have revealed a complex landscape of genetic, epigenetic, and microenvironmental determinants that shape gliomagenesis. These insights raise an essential question: if we now possess a detailed understanding of the molecular phenotype of glioma-initiating cells, why do effective targeted therapies remain elusive?

**Figure 2:**
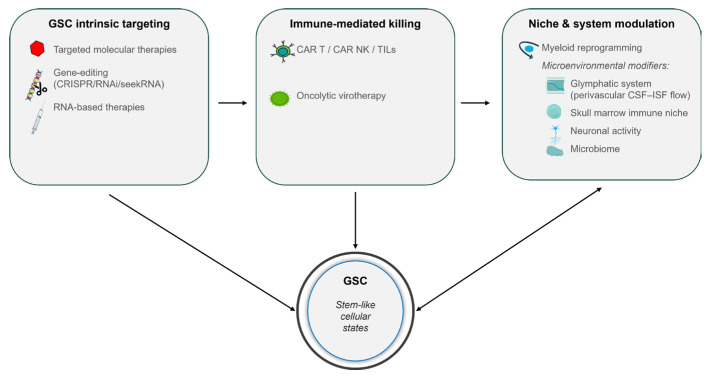
Therapeutic convergence on glioblastoma stem-like cellular states.

Meaningful progress is being made. Standard-of-care DNA-alkylating agents such as temozolomide remain foundational, and targeted therapies directed at oncogenic pathways—including EGFR, STAT3, and Wnt—continue to evolve. Novel EGFR variants such as EGFRx maintain glioblastoma stemness through STAT5 activation [36], while STAT3 remains essential for proliferation and multipotency [37]. Wnt-mediated endothelial-to-mesenchymal transitions contribute to chemoresistance and tumor progression [38]. Importantly, these pathways are not merely generic oncogenic drivers; they are central regulators of glioblastoma stem cell (GSC) self-renewal, lineage plasticity, and apoptotic resistance. Consequently, pathway-directed therapies are increasingly informed by the biology of stem-like cellular states rather than by mutational status alone. Contemporary therapeutic strategies therefore converge on GSC states through complementary mechanisms targeting intrinsic survival programs, immune-mediated cytotoxicity and niche- and system-level modulators (Figure 2).

Contemporary precision therapies for glioblastoma increasingly target stem-like cellular states that sustain tumor propagation, cellular plasticity and therapeutic resistance. GSC-intrinsic strategies include targeted molecular therapies, RNA-based platforms and emerging gene-editing approaches that disrupt self-renewal and survival programs. Immune-mediated killing is achieved through cellular immunotherapies, including chimeric antigen receptor (CAR) T cells, CAR natural killer (NK) cells and tumor-infiltrating lymphocytes (TILs), as well as oncolytic virotherapy. In parallel, niche- and system-level modulation—including myeloid reprogramming, glymphatic transport (perivascular cerebrospinal fluid–interstitial fluid flow), skull marrow immune niches, neuronal activity, and microbiome-associated influences—shapes GSC maintenance and therapeutic responsiveness. Together, these complementary approaches emphasize a state-centric model of therapeutic intervention in glioblastoma.

Within this framework, therapeutic innovation in glioblastoma increasingly emphasizes coordinated disruption of stem-like cellular states through direct targeting of GSC-intrinsic programs, immune-mediated killing and modulation of permissive niches and systemic regulators.

In parallel, immunotherapy platforms have expanded considerably, including antibody–drug conjugates (ADCs) targeting EGFR, HER2, and other glioma-associated antigens offering selective cytotoxic delivery [39]. RNA-based immunotherapies—including mRNA vaccines, miRNA modulators, and RNA aggregates—are rapidly advancing. RNA aggregates, in particular, can engage innate danger-signal pathways to drive potent antitumor immunity [40]. Engineered extracellular vesicles, such as TRAIL-loaded exosomes derived from induced neural stem cells, provide a biocompatible delivery system for RNA and protein therapeutics [41].

Advances in base editing and CRISPR–Cas9 technologies have progressed from in vitro applications to in vivo gene correction and oncogene disruption in glioma models [42]. Hybrid guide RNAs improve the specificity and efficiency of adenine base editing [43], while emerging systems such as SeekRNA offer programmable RNA-guided editing with reduced off-target activity [44]. These platforms allow for direct in vivo modification of oncogenic drivers or restoration of tumor suppressors, offering unprecedented precision in targeting the molecular lesions that sustain GSCs.

Adoptive cellular therapies also continue to advance. CAR T cells targeting IL13Rα2, EGFRvIII, HER2, and GD2 have demonstrated safety and early signs of efficacy in glioblastoma clinical trials [45,46,47]. CAR NK cells offer additional advantages, including reduced toxicity and the possibility of universal donor-derived products. Tumor-infiltrating lymphocyte (TIL) therapies are gaining traction as single-cell profiling reveals resident tumor-reactive lymphocyte subsets. Despite limited efficacy in unselected GBM populations, immune checkpoint inhibitors remain central to immunotherapy research; PD-1, PD-L1, CTLA-4, TIM-3, and LAG-3 blockade may be effective when combined with vaccines, oncolytic viruses, or myeloid-modulating therapies [48,49].

Oncolytic virotherapy—including adenovirus DNX-2401, poliovirus chimera PVSRIPO, and HSV-based vectors—induces immunogenic tumor cell death, reshapes the tumor microenvironment, and synergizes with ICIs [50,51]. Given that glioblastoma is dominated by immunosuppressive myeloid cells, strategies targeting CSF1R, CD47–SIRPα, TREM2, and other myeloid checkpoints aim to reprogram these populations toward antitumor phenotypes [52].

With single-cell sequencing, spatial transcriptomics, and lineage tracing in their prime, a detailed catalog is emerging of both normal neurogenic and aberrantly neurogenic glioma-like states. These datasets reveal how adult brain stem cells, when forced back into proliferative states—whether through aging, injury, inflammation, or spontaneous mutation—become vulnerable to oncogenic transformation. Additional insights into activity-dependent tumorigenesis [53], the influence of the glymphatic system on inflammatory tone [54], and the discovery of skull-marrow immune niches containing complete lymphoid and myeloid lineages [55,56] further expand the therapeutic landscape. Newly identified tertiary-lymphoid-like structures connected to the tumor via meningeal bridges [56] represent novel targets for immunomodulation and drug delivery.

## 5. Future Directions: Toward Precision and Personalization

Recurrent gliomas exemplify the evolutionary capacity of malignant systems, frequently undergoing profound genotypic and phenotypic remodeling marked by the re-emergence of stem cell-like states [57] and the adoption of noncanonical modes of intercellular communication. Transcellular transfer of organelles—including mitochondria and RNA—between tumor cells and stromal elements [58] has emerged as a potent mechanism by which gliomas enhance metabolic fitness, evade therapy, and increase tumorigenicity. These processes reinforce the view of gliomas as adaptive, cooperative cellular ecosystems rather than collections of independently evolving clones.

These adaptive, spatially structured stem-like states generate levels of biological complexity that exceed the capacity of linear biomarker-based approaches, providing a strong rationale for AI-driven integrative models in glioblastoma.

Beyond tumor-intrinsic programs, systemic modifiers of gliomagenesis are gaining increasing attention. The gut and brain microbiomes influence immune tone, metabolism and therapeutic responsiveness, suggesting that nutritional status and microbial composition may shape both tumor initiation and treatment outcomes [59]. Integrating systemic physiology with tumor and microenvironmental biology broadens the conceptual framework of neuro-oncology and suggests new opportunities for metabolic, immune and microbiome-directed interventions.

Artificial intelligence provides a unifying framework to translate this multiscale complexity into actionable therapeutic strategies. Machine learning approaches integrating multi-omic, spatial, and imaging data are increasingly capable of identifying druggable dependencies, modeling cellular states, and stratifying patients based on evolving molecular and immunological features [60]. Active learning frameworks coupled with transcriptomic profiling and advances in spatial omics and high-resolution imaging further enable region-specific analysis of tumor architecture, lineage relationships and immune infiltration, supporting rational, adaptive deployment of combination therapies [61,62].

Innovations in data science will increasingly determine the timing, selection, and combination of immune-based, molecular, and cell-directed therapies for glioblastoma and related cancer stem cell driven diseases.

## Data Availability

No new data were created or analyzed in this study.
